# The Prevention of Tooth Decay1Read December 18, 1906, at the monthly meeting of the Eighth District Dental Society, New York.

**Published:** 1907-02

**Authors:** Frank W. Low

**Affiliations:** Buffalo, N. Y.


### The Prevention of Tooth Decay.[1]

By FRANK W. LOW, Buffalo, N. Y.

THAT the teeth of men are immune from decay because of the habit of smoking tobacco, is a saying almost as old as smoking tobacco itself. The observation of many generations has made the saying pass current to such an extent that, viewed as a prevention, it might rightly be classed with other empirical medication. Recent laboratory investigation, however, leads pointedly to the belief that the saying is true, and the following observations furnish the theory upon which the proving rests.

All normal human saliva has in it a certain portion of potassium sulphocyanate,[2] but in mouths where rapid and general decay of the teeth is progressing, it is invariably found that potassium sulphocyanate is conspicuous for its absence. Saliva from the mouths of smokers invariably has a generous proving of potassium sulphocyanate. More or less decay occurs in the human teeth in any event, because the bacteria of decay find lodgement wherever there is roughened and defective enamel, such as, for example, the crevices formed in the sulci resulting from the infolding of the enamel on occlusial surfaces between cusps.

According to the theory of Black[3]—wherever decay occurs elsewhere on the teeth it is because the bacteria themselves, under favorable conditions, have the ability to form small films or plaques of a gelatinoid character, which float about in the saliva until they become glued upon some accessible surface of the tooth, where they remain undisturbed for a sufficient time to allow the bacteria imbedded in them to corrode the enamel surface. This form of decay is most rapid and does much greater mischief than any other. The theory that accounts for the absence of these plaques in the saliva of individuals who have marked provings of potassium sulphocyanate, rests on the fact that potassium sulphocyanate is a powerful solvent of all gelatinous substances.

After exhaustive experiments covering a period of more than three years, the Committee on Scientific Research of the New York State Dental Society has come to the conclusion that this reasoning is correct and is recommending the following prescription to be given on alternate weeks covering indefinite

---

1. Read December 18, 1906, at the monthly meeting of the Eighth District Dental Society, New York.

2. " The presence of sulphocyanids in mixed saliva is considered normal by certain authors."—Sialo-Semeiology by Dr. Joseph P. Michaels, Paris, France.

3. G. V. Black, D. D. S., Dean of the North Western University, Chicago, Ill.

periods—namely, until such time as the saliva continues to show the presence of potassium sulphocyanate without the aid of medication:

℞

Potasii Sulphocyanate[4] gr. xxxij
Aq. Cinnamon ℥ j
Syr. Simplex qs—to make—℥ iv
Sig. Teaspoonful at bedtime.

It is believed that the quantity of potassium sulphocyanate, which will begin immediately to appear in saliva of patients to whom the above prescription is administered, is sufficient to prevent bacteria from forming the gelatinoid substance of which the plaques are made and, since without this they cannot attach themselves to smooth surfaces, the worst form of rapid decay will consequently be prevented.

52 North Pearl Street.

4. For the greater convenience of administration Parke, Davis & Co. are making a compressed tablet containing ½ grain Potassium Sulphocyanate. 2 tablets may be taken at bedtime in place of above prescription. 1 tablet should be administered in place of 2 to children.

